# Effect of biofertilizers on leaf yield, nitrate amount, mineral content and antioxidants of basil (*Ocimum basilicum* L.) in a floating culture

**DOI:** 10.1038/s41598-022-24799-x

**Published:** 2022-12-03

**Authors:** Hayriye Yildiz Dasgan, Abdullah Aldiyab, Farah Elgudayem, Boran Ikiz, Nazim S. Gruda

**Affiliations:** 1 Department of Horticulture Science, Faculty of Agriculture, University of Cukurova, 01330 Adana, Turkey; 2 Laboratory of Ecosystems and Biodiversity in Arid Areas in Tunisia, Department of Life Sciences, Faculty of Sciences of Sfax, University of Sfax, 3000 Sfax, Tunisia; 3 Institute of Plant Sciences and Resource Conservation, Division of Horticultural Sciences, University of Bonn, Bonn, Germany

**Keywords:** Plant sciences, Environmental sciences

## Abstract

Hydroponics is one of the systems in agriculture which reinforce productivity by controlling environmental and growing conditions. In this study, we evaluated the effect of three bio-fertilizers, namely bacteria, micro-algae, and mycorrhiza, on basil leaf yield and quality (*Ocimum basilicum* L.) in a floating culture system. Soil has rich amounts of beneficial microorganisms, supporting plant nutrition, producing phytohormones, controlling phytopathogens, and improving soil structure. However, soilless culture usually contains no beneficial microorganisms if we do not include them in the system. This study aims to evaluate the response of three bio-fertilizers where mineral fertilizers are reduced by 50%. Considering the total harvest data, bacteria, mycorrhiza, and micro-algae treatments increased basil yield compared to 50% control by about 18.94%, 13.94%, and 5.72%, respectively. The maximum total yield and leaf area were recorded using bacteria with 2744 g m^−2^ and 1528 cm^2^ plant^−1^. Plants with mycorrhiza achieved the highest number of leaves and branches, with 94.3 leaves plant^−1^ and 24.50 branches plant^−1^, respectively. It was observed that this bio-fertilizer increased the formation of lateral branches in the basil plant without thickening its stems. In addition, bacteria and mycorrhiza induced the highest percentage of dry matter and total soluble solids. The effect of bio-fertilizers on basil leaf EC and pH was insignificant for all the treatments at different harvest periods (*p* < 0.05). Using bio-fertilizers enhanced the intake of nutrients N (nitrogen), P (phosphorus), K (potassium), Ca (calcium), Mg (magnesium), Fe (iron), Mn (manganese), Zn (zinc), and Cu (copper). Using bio-fertilizers represents a promising and environmentally friendly approach to increasing crop yields and ameliorating quality and antioxidant compounds with fewer resources. An application of bio-fertilizers in hydroponic cultivation of basil cv. ‘Dino’ reduced the need for mineral fertilizers. At the same time, bio-fertilizers affected an increased plant yield and improved product quality. Furthermore, the bacteria had a pronounced enhancing effect on the increase of phenol and flavonoids in the leaves of basil plants.

## Introduction

Diminishing arable land, increasing urbanization, water limitation, and climate change enforce pressure on agricultural production. The current world population is predicted to increase to 10 billion by 2050. In addition, the city’s living rate is expected to increase from 50 to 70% in the 2030s^1^. Furthermore, the increasing effects of climate change, such as drought, salinity, floods, and sudden temperature changes, negatively affect conventional agriculture. Soilless culture can reduce the impact of these factors and offer a solution that ensures sustainable food security in the future.

Soilless culture systems mainly operate in controlled environmental conditions (greenhouse or plant factory) and increase water use efficiency, especially in closed systems with a recirculating water/nutrient solution that recaptures the drain water for reuse^1^. The main advantage of soilless cultures is that plants are grown in a controlled environment. Soilless cultivation improves control of the growing medium and avoids problems with irrigation and maintaining proper nutrient concentrations. Hence, good control of the plant growth and better quality crops compared to traditional production in soil.

The hydroponic system, one soilless culture system, is defined as the cultivation of plants without the physical support of a substrate or soil for the roots, usually provided with a nutrient solution^2,3^. Floating is a kind of hydroponic in which the plant roots are immersed in an aerated nutrient solution. The solution comprises water and fertilizers, precisely dosed to obtain the appropriate concentration of macro- and microelements necessary for plant growth^4^. In large-scale-cultivation, the system operates in a closed circuit: the nutrient solution is pumped, recycled, and renewed regularly. The recirculation allows the aeration of the nutrient solution, where its pH and electro-conductivity (EC) are continuously monitored^5^. In hydroponic systems, the smaller planting area is effectively used. As a result, it requires less water consumption.

Furthermore, this culture is also successfully used in some regions with poor soil conditions or regions characterized by drought and famine^2,6^. The hydroponic growing systems are predominantly used to produce leafy vegetables, such as lettuce, basil, spinach, and rocket salat^2^. Basil is the second most widely grown leaf vegetable after lettuce.

Basil (*Ocimum basilicum* L.) is an important plant with essential oils, polyphenols, phenolics and flavonoids grown for food, pharmaceutical and cosmetic purposes. Among the aromatic plants in which its consummation gradually increases is the basil, a plant from the *Lamiaceae* family, genus *Ocimum*^7^. It usually grows in tropical and subtropical areas and is widely used in the human diet as a fresh vegetable or spice^8^.

Indeed, it stands out with its strong and unique aroma in meat, vegetable dishes, salads, sauce, herbal tea and other culinary uses. However, its primary culinary interest in this plant has increased in value, as it is not only an aromatic plant but also a medicinal one, widely used in health treatments^9^. It must be noted that some vegetables, including basil, could contain high nitrate levels^10^. Most of the vegetables are fresh products that are not processed. Thus, it is imperative to maximize the product quality of vegetables through appropriate growing techniques^11^. Soilless culture techniques under controlled environments (greenhouse or plant factory) through plant nutrition, LED (Light Emitting Diode) lighting, and biofertilisers positively affect the crop nutritional quality.

Nowadays, many organic or mineral products spread over the earth increase the yield and improve the plant’s nutrition^12^. Organic fertilizers differ from mineral ones because they are not directly assimilable by the plant. Thus, microorganisms must decompose these compounds before their use^13^. However, organic fertilizers are usually natural. Hence they are environmentally friendly, characterized by the absence of chemicals^14^ and contain vital microorganisms. Soil has rich amounts of beneficial microorganisms, supporting plant nutrition, producing phytohormones, controlling phytopathogens, and improving soil structure. However, soilless culture usually contains no beneficial microorganisms if we do not include them in the system. Therefore, implementing biofertilizers in soilless culture can be a great and eco-friendly alternative to enrich plant nutrition and reduce the use of chemical fertilizers.

Bio-fertilizers are preparations containing living cells or latent cells of efficient strains of microorganisms that help crop plants uptake nutrients^15^. Recently, bio-fertilizers became popular in soilless growing systems due to their environmentally approaches, plant nutrition efficiency and decreasing mineral fertilisation cost. Moreover, they induce plant resistance to biotic and abiotic stress factors and increase plant growth and yield^15^. One of the green algae species of *Chlorella vulgaris* is rich source of proteins, lipids, carbohydrates, pigments, and metabolites with antioxidant properties. The use of *Chlorella vulgaris* in conventional agriculture is well known. However, its use as a live algal cell in hydroponics is minimal.

Plant growth-promoting rhizobacteria (PGPR), most of their strains belonging to species of *Pseudomonas* or *Bacillus*, can enhance plant growth capacity by increasing seed emergency, plant weight, and yield. In addition, some of the PGPR induce systemic resistance to fungi, bacteria, viruses, and in some cases, nematodes^27^.

Arbuscular mycorrhizal (AM) fungi occur in all soil types and commonly colonize the roots of many plant species. These fungi can increase plant growth and reproduction by enhancing the uptake of nutrients. AM fungi can also benefit plants by stimulating growth-regulating substances, increasing photosynthesis, improving tolerance against stresses and increasing pest resistance^23^.

This study aims to evaluate the response of the bio-fertilizers where mineral fertilizers are reduced by 50%. We investigated growth parameters, nitrate amount, basil leaf nutrients and antioxidant content in a floating system. To our knowledge, this is the first study to include vital beneficial microorganisms in basil growing in the floating water culture system.

## Material and methods

### Plant material and growing conditions of basil in a floating system

The present study was conducted in a glasshouse between the spring and the summer of 2019 at the University of Cukurova, Adana, Turkiye (36°59′N, 35°18′E, 20 m above sea level). Basil seeds (*Ocimum basilicum* L. ‘Dino’) were provided by Enza Zaden seed company. Seeds were sown with mycorrhiza in mini rockwool cubes. After their transfer to a hydroponic system, other bio-fertilisers, such as bacteria and micro-algae, were applied for 60 days.

Basil plants were grown using the ‘floating culture’ method, in which the plant roots were immersed in the aerated nutrient solution in plastic tanks with a total volume of 50 L (55 × 105 cm). The water level in the containers was maintained constant, and the nutrient solution was changed every ten days. The distance between the rows of basil plants was 15 × 15 cm, with a plant density of 44.44 plant m^−2^. During the trial, the pH and EC of the nutrient solution in the hydroponic system were measured daily. The pH was kept between 5.5 and 6.0, and the EC was fixed at 1.5 and 2.0 µS cm^−1^. These pH and EC trend followed the findings of Wortman^31^.

Two stock solutions were used: stock A (Potassium nitrate, calcium nitrate, ammonium nitrate, and Fe-EDDHA) and stock B (potassium sulfate, mono-potassium sulfate, magnesium sulfate, microelements, zinc sulfate, boric acid, manganese sulfate, and ammonium molybdate).

The nutrient solution consists of nitrogen (N) 160 to 200 ppm, phosphorus (P) 30–40 ppm, potassium (K) 220–250 ppm, calcium (Ca) 140–160 ppm, magnesium (Mg) 40–50 ppm, Iron (Fe) 2.5–5.0 ppm, manganese (Mn) 0.25–0.4, Boron (B) 0.25–0.40 ppm, Zinc (Zn) 0.20–0.50 ppm, copper (Cu) 0.02–0.05 ppm, and molybdenum (Mo) 0.04–0.07 ppm. Treatments were performed by substituting the bio-fertilizers for reduced mineral fertilizers. In the study, there were five treatments described as follows:

1. Full strength nutrient solution was called 100% mineral fertilization (control-1),
2. 50% mineral fertilization (control-2),
3. 50% mineral fertilization with micro-algae,
4. 50% mineral fertilization with bacteria,
5. 50% mineral fertilization with mycorrhiza.

This study complies with relevant institutional, national, and international guidelines and legislation.

### Experimental design

Randomized blocks experimental design, with 15 plants in each replication (60 plants per treatment) was used. Glasshouses represent controlled environmental systems regarding climatic conditions, such as heating, cooling, relative humidity, CO_2_ fertilisation and lighting. Therefore we prefered a randomised blocks experimental design. Thus for one recurrence was 0.3375 m^2^. Seeds were sown on March 21, and after 40 days, when the seedlings had three true leaves, they were transplanted into the floating system. The nutrient solution renewal was stopped entirely ten days before the latest harvest. The mean temperature in the greenhouse was 26 °C (min. 16.3 °C and max. 33.5 °C), and the relative humidity was 57%. Basil was harvested four times by cutting upper young leaves on May 20, June 2, June 16 and June 30. Fresh leaves per plant were weighed in each harvest to provide the total leaf yield.

### Bio-fertilizers

‘*Chlorella vulgaris*’ strain of micro-algae was used in 2 × 10^7^ mL^−1^ concentration. The inoculation was diluted 40 times before its use^15^. For 1 L nutrient solution, 25 mL of algae was added from the 2 × 10^7^ mL^−1^ concentration.

Three bacterial species (*Bacillus subtilis*, *Bacillus megaterium*, and *Pseudomonas fluorescens*) were obtained from NGB (Next Generation Biotechnology) Company with a trading name ‘Rhizofill’. A mixture of 50 mL of the colony (1 × 10^9^ mL^−1^) was added every ten days in a 50 L nutrient solution.

The commercial mycorrhiza was obtained from ERS (Endo Root Soluble) Company (Bioglobal). The mixture was composed of *Glomus intraradices*, *Glomus aggregatum*, *Glomus mosseae*, *Glomus clarum*, *Glomus monosporus*, *Glomus deserticola*, *Glomus brasilianum*, *Glomus etunicatum*, and *Gigaspora margarita (*1 × 10^4^ w:w). During seed planting, 1000 spores plant^-1^ were used.

### Yield, yield components and quality of basil leaves

#### Measurement of basil yield, leaf area, and its number at four harvest

The cumulative yield of basil is expressed as g m^−2^ for four harvests. At the same time, the number of leaves per plant was recorded. Afterwards, the leaf area was determined by leaf area meter (Li-3100, LICOR, Lincoln, NE, USA) and indicated as cm^2^ plant^−1^.

#### Evaluation of the number of branches, stem diameter, dry matter and its solubility

The number of branches was calculated at the last harvest, and the stem diameter was measured with a digital calliper. Dry weight (DW) was obtained in a forced-air oven at 70 °C until constant weight. Dry matter (DM) was measured by weighting fresh (FW) and dried basil material and expressed in percentage (DM = 100 × DW/FW). Besides, the total soluble solution (soluble dry matter) was measured with a digital refractometer and was expressed in percentages.

#### Measurement of EC, pH, and nitrate amount of basil leaf

The electrical conductivity (EC) and basil leaf pH were determined at the last harvest. The nitrate analysis was performed calorimetrically at 410 nm according to Cataldo et al.^16^ using the salicylic acid method and their concentrations were expressed as µg NO_3_^–^N per dry weight (ppm).

#### Determination of micro and macro elements in basil leaf

Macro and microelements analyzes were carried out to reveal the effects of different treatments on nutrition in basil plants. For nitrogen (N), phosphorus (P), potassium (K), magnesium (Mg), copper (Cu), and zinc (Zn) analysis, the plant material was washed with distilled water three times. Then, it was dried in an oven at 65 °C for 48 h. The dried samples were ground in the mill and then kept at 550 °C for eight hours, and the ash formed was dissolved in 3.3% HCl. An atomic absorption spectrometer read the microelements (Fe, Mn, Zn, and Cu) from the filtrated solution. While for the macro elements (K, Ca and Mg), 1 mL of the filtrated solution was added to 19 mL of distilled water. According to Barton and Kjeldahl methods, phosphorus and nitrogen analyses were carried out by spectrophotometer, respectively^17,18^.

#### Determination of total phenolic substance, flavonoids and vitamin C leaf

Total phenolics were determined by modifying the spectrophotometric method described by Spanos and Wrolstad^19^. The readings were calculated using the absorbance value read at 765 nm wavelength in the spectrophotometer (UV-1700 PharmoSpec Shimadzu, Japan) and the calibration curve prepared with gallic acid. Total flavonoids were analyzed using a spectrophotometer set to 415 nm, following a method developed by Quettier-Deleu et al.^20^. The total amount of flavonoid substances was calculated using the calibration curve created by the rutin. Vitamin C was determined by the modified method of Elgailani et al.^21^. Basil leaves were blended with a high-speed blender, and 5 mL basil extract was mixed with 45 ml 0.4% oxalic acid and then filtered. One milliliter filtrate and 9 ml 2,6-dichlorophenolindo-phenol sodium salt were mixed, and the transmittance value was measured at 520 nm using UV spectrophotometer.

### Statistical analysis

Data were exposed to ANOVA test using SAS-JUMP/7. In addition, the Fisher’s LSD test was used to compare the averages at a 5% significance level.

## Results and discussion

Nowadays, there is a request for food production to operate sustainably and not at the expense of human health, the environment and the life of future generations. Therefore, the call of the time is to develop new agricultural systems that produce more with fewer resources, e.g., phytosanitary products and fertilisers. Using bio-fertilizers represents a promising and environmentally friendly approach to increasing crop yields and ameliorating quality and antioxidant compounds with fewer resources. This fits very well with the UN Sustainability Goal 12: “Ensure sustainable consumption and production patterns”.

### Effect of bio-fertilizers on yield, leaf area, and number at four harvest

Several freah leaf harvests can be conducted for basil during one vegetation period. Compared to the 50% control, in the first harvest, there was no significant difference in basil leaf yield. Mycorrhiza treatment improved basil yield by 214.48% in the second harvest compared to the 50% control. Bacteria raised basil yield only by 17%, compared with the 50% control in this harvest. The bacteria and micro-algae treatments increase basil yield in the third harvest. In the last harvest, bacteria induced a yield of about 19.20% when we compared it to the 50% control. Considering the total harvest data, bacteria, mycorrhiza, and micro-algae treatments increased basil yield compared to 50% control by about 18.94%, 13.94%, and 5.72%, respectively (Table 1). The increase in the yield in the second and third harvests using mycorrhiza was less than the yield obtained in the last harvest. This can be explained by the increase in the temperature at the end of June in the glasshouse. Most likely, the temperature up to 30 °C helped to increase the activity of bacteria, exceeding the effect of mycorrhiza^22,23^.

**Table 1:** Leaf yield (fresh) of basil plants grown with different bio-fertilizers at four harvest times and total (g m^−2^).

Treatments	May 20	June 2	June 16	June 30	Total harvest
100% mineral fertilizers	195^a^	304^b^	680^a^	2522^a^	3702^a^
50% mineral fertilizers	160^ab^	145^cd^	372^b^	1683^c^	2307^d^
50% mineral fertilizers + micro-algae	124^bc^	99^d^	494^b^	1722^c^	2439^cd^
50% mineral fertilizers + bacteria	109^c^	170^b^	477^b^	1989^b^	2744^b^
50% mineral fertilizers + mycorrhiza	155^ab^	456^a^	376^b^	1630^c^	2616^bc^
LSD_0.05_	39.32	56.50	151.33	110	176.02
*p*-value	0.4022	< 0.0001	0.5253	< 0.0001	< 0.0001

*LSD* the least significant difference between the means (*p* < 0.05). There is no significant difference between means with the same letter in the same column

In contrast, adding the micro-algae to a 50% nutrient solution was not very effective in yield. The lack of mineral fertilizers may have created an unfavourable environment for microalgae to live and multiply^24^. The basil plant and *Chlorella vulgaris* would use the mineral nutrients for photosynthese. Perhaps, there may be competition for mineral fertilizers between basil and microalgae.

The inoculation by PGPRs and mycorrhiza may encourage the production of biologically active substances such as phytohormones, amino acids and water-soluble vitamins. Some activating hormones, which play an essential role in plant growth and the bio-fertilization, may increase the contents of IAA (Indole Acetic Acid), Cytokinins and GA_3_ (Giberallic Acid)^25^. These phytohormones enable plant cell growth and division and the extension of roots and effect the hormonal balance of plants^27^. It is reported that the application of bio-fertilizers had stimulating effects on plants by hormones, nitrogen fixation, phosphate solubilization, and siderophore production and helped reduce the use of chemical mineral fertilizers and improved plant growth and productivity^26,27^.

The best basil leaf area was recorded in the first harvest when we used 50% mycorrhiza. However, 50% of bacteria showed the highest leaf area in the second and the last harvest. In the third harvest, it was observed that 50% micro-algae treatment exhibited the best leaf area. For the total harvest, 50% bacteria and 50% mycorrhiza shows the highest total area, respectively, while micro-algae manifest the less area (Table 2). Based on the previous data, bacteria, mycorrhiza, and micro-algae show an increased rate of fresh leaf weight, but in terms of area, it was not proportional, which can be explained by the increase in the thickness of leaves^28,29^.

**Table 2:** Leaf area of basil, obtained with different bio-fertilizers at four harvest times and total (cm^2^ plant^−1^).

Treatments	May 20	June 2	June 16	June 30	Total harvest
100% mineral fertilizers	148	243^ab^	321^a^	976^a^	1688^a^
50% mineral fertilizers	123	207^bc^	272^b^	771^c^	1372^c^
50% mineral fertilizers + micro-algae	121	189^c^	298^ab^	648^d^	1256^d^
50% mineral fertilizers + bacteria	117	280^a^	193^c^	938^a^	1528^b^
50% mineral fertilizers + mycorrhiza	134	234^b^	227^c^	837^b^	1431^c^
LSD_0.05_	ns	42.037	20.394	29.145	41.475
*p*-value	0.8428	0.5850	0.9186	< 0.0001	0.2807

*LSD* least significant difference between the means (*p* < 0.05); ns: non-significant. There is no significant difference between means with the same letter in the same column.

Compared to 50% control, for all harvest periods, bio-fertilizers harm the number of basil leaves with a decrease of 23.67% in bacteria, 19.67 in micro-algae, and 10.6% in mycorrhiza treatment (Table 3). While the number of leaves decreased in biofertilizer compared to 50% mineral fertilizer, leaf weight increased. The effect of biofertilizers; It can be explained as fewer but thicker and heavier leaves. The decrease can be interpreted by the increase in the yield and the thickness of leaves numbers^30^.

**Table 3:** The total number of leaves obtained at four harvest times and total (pieces plant^−1^).

Treatments	May 20	June 2	June 16	June 30	Total harvest
100% mineral fertilizers	9.6^a^	11.0	10.7^a^	78.9^a^	110.1^a^
50% mineral fertilizers	9.2^ab^	11.3	10.0^ab^	74.8^ab^	105.2^ab^
50% mineral fertilizers + micro-algae	7.3^bc^	10.1	9.0^bc^	58.1^c^	84.5^cd^
50% mineral fertilizers + bacteria	6.2^c^	10.6	7.6^d^	56.0^c^	80.3^d^
50% mineral fertilizers + mycorrhiza	7.7^abc^	11.4	8.7^c^	66.5^c^	94.3^bc^
LDS_0.05_	1.021	ns	0.502	5.502	5.653
*p*-value	0.5573	0.652	0.0110	0.3950	0.2924

*LSD* the least significant difference between the means (*p* < 0.05), *ns* non-significant. There is no significant difference between means with the same letter in the same column

### Effect of bio-fertilizers on pH, EC, and solubility of dry matter of basil leaf

As expected, the basil EC of 7.12 dS m^−1^ was the highest in 100% mineral fertilizer. In applications where mineral fertilizers were reduced by 50%, and bio-fertilizers were applied, EC varied between 5.98 and 6.69. Similarly, the highest pH of 6.55 basil leaves was observed in 100% mineral fertilizer application. pH values of bio-fertilizer applicated basil leaf varied between 6.11 and 6.20. (Table 4). The bioferilizer lowered basil leaf pH. The biofertiliser may have stimulated the production of some organic acids that would lower the pH in the basil leaves.

**Table 4:** Effect of bio-fertilizers on some leaf chemical properties.

Treatments	EC(dS m^−1^)	pH	SDM (%)
100% mineral fertilizers	7.12^a^	6.55^a^	1.75^b^
50% mineral fertilizers	5.98^b^	6.20^b^	1.18^c^
50% mineral fertilizers + micro-algae	6.57^ab^	6.11^b^	1.62^b^
50% mineral fertilizers + bacteria	6.69^a^	6.19^b^	1.67^b^
50% mineral fertilizers + mycorrhiza	6.48^ab^	6.19^b^	1.95^a^
LSD 0.05	0.650	0.008	0.015
*p*-value	0.4250	0.0001	< 0.0001

*LSD* the least significant difference between the means (*p* < 0.05), *ns* non-significant, *SDM* Soluble dry matter. There is no significant difference between means with the same letter in the same column

Compared to the 50% control, the water-soluble dry matter of basil (SDM) shows a highly significant difference where it varies between 1.95 and 1.18%. Micro-algae, bacteria, and mycorrhiza increased the SDM by 37.6%, 40.6%, and 65.9%, respectively (Table 4). Biofertilizers can benefit plants by enhancing the uptake of nutrients, stimulating growth, regulating substances, and increasing photosynthesis. Therefore water-soluble dry matter can be significantly increased compared to 50% mineral fertilisers. Maboko et al.^32^ reported that mycorrhiza inoculation in the soilless medium increased SDM.

### Effect of bio-fertilizers on stem diameter, dry matter, and number of branches in basil

The stem diameter was decreased from the thickest to the thinnest with the treatment of mycorrhiza, bacteria, and microalgae, respectively. Moncada et al.^33^ stated that rhizobacteria *(Bacillus* spp.) increase basil thinnest in a hydroponic system (Table 5).

**Table 5:** Effect of bio-fertilizers on stem diameter, dry matter, and the number of branches.

Treatments	SD (mm)	DM (%)	NB (piece plant^−1^)
100% mineral fertilizers	4.000^a^	8.42^c^	26.10^a^
50% mineral fertilizers	3.695^b^	9.38^b^	17.00^e^
50% mineral fertilizers + micro-algae	3.235^e^	9.21^b^	20.75^c^
50% mineral fertilizers + bacteria	3.400^d^	10.14^a^	18.40^d^
50% mineral fertilizers + mycorrhiza	3.553^c^	9.98^a^	24.50^b^
LSD_0.05_	0.329	0.868	2.123
*p*-value	< 0.0001	< 0.0001	< 0.0001

*LSD* the least significant difference between the means (*p* < 0.05), *ns* non-significant, *SD* Stem diameter, *DM* Dry matter, *NB* Number of branches. There is no significant difference between means with the same letter in the same column

Using bacteria further increased the dry matter (DM) by 8.10%. Also, when mycorrhiza was added to the 50% nutrient solution DM increased by 6.40%. Plant growth functions, stomatal conductance, leaf water potential, relative water content, PSII efficiency, mineral nutrient availability and CO_2_ assimilation are positively affected by mycorrhizal inoculation^34,36^. Therefore, inoculation of mycorrhiza improves the accumulation of dry matter. In contrast, using micro-algae leads to a decrease in DM by 1.81% (Table 5). According to^36^, the increase in DM ameliorates the basil leaves’ quality and aroma. The number of branches increased using *Glomus* spp. by 44%, compared to 50% control, which is close to 100% control (Table 5). Plant symbiotic relationship with mycorrhiza may improve root size and efficiency, hence, leaf area index and biomass^35^. The number of branches is crucial for the yield^37^. The more efficiency of photosynthesis process and increments of carbohydrates supply to basil leaf can explain improvements of branche’s number and leaf dry matter.

### Effect of bio-fertilizers on nitrate amount in basil leaf

**Figure 1:**
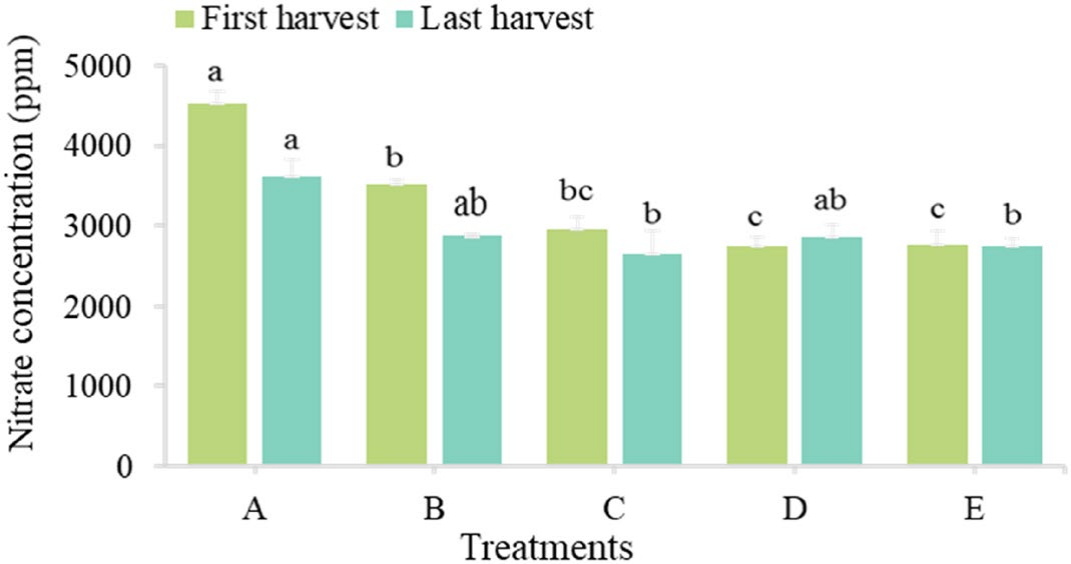
Effect of bio-fertilizers on nitrate amount of basil grown in the hydroponic system (ppm). 100% Mineral fertilizers (control, A), 50% mineral fertilizers (control, B), 50% mineral fertilizers + micro-algae (C), 50% mineral fertilizers + bacteria (D), 50% mineral fertilizers + mycorrhiza (E).

Nitrate accumulation in leafy vegetables is affected by nitrogen amount and source, light intensity, temperature and genotypes.The measurement of nitrate amount was determined in the first and last harvests. In the two harvests, compared with the 50% control, nitrate levels were low for all treatments. In the first harvest, a decrease of 16.3%, 22.9%, and 21.5% for micro-algae, bacteria, and mycorrhiza treatments, was shown, respectively. While in the last harvest, the decrease in nitrate amount was about 7.8%, 0.2%, and 4.6% for micro-algae, bacteria, and mycorrhiza, respectively (Fig. 1). The results are due to effect of bio-fertilizers with their low N property may have in reducing the nitrate content in basil leaf compared with mineral fertilizer. As the harvest progresses, nitrate degradation in basil seems to have occurred more since the light intensity increased from May to the end of June. Rouphael et al.^38^ found that the nitrate amount decreased from autumn to summer. Moreover, Orsini and de Pascale^39^ found that the nitrate amount in basil grown in the hydroponic system and 50% shade was more than the basil exposed to light.

### Effect of bio-fertilizers on macro elements in basil leaf

**Figure 2:**
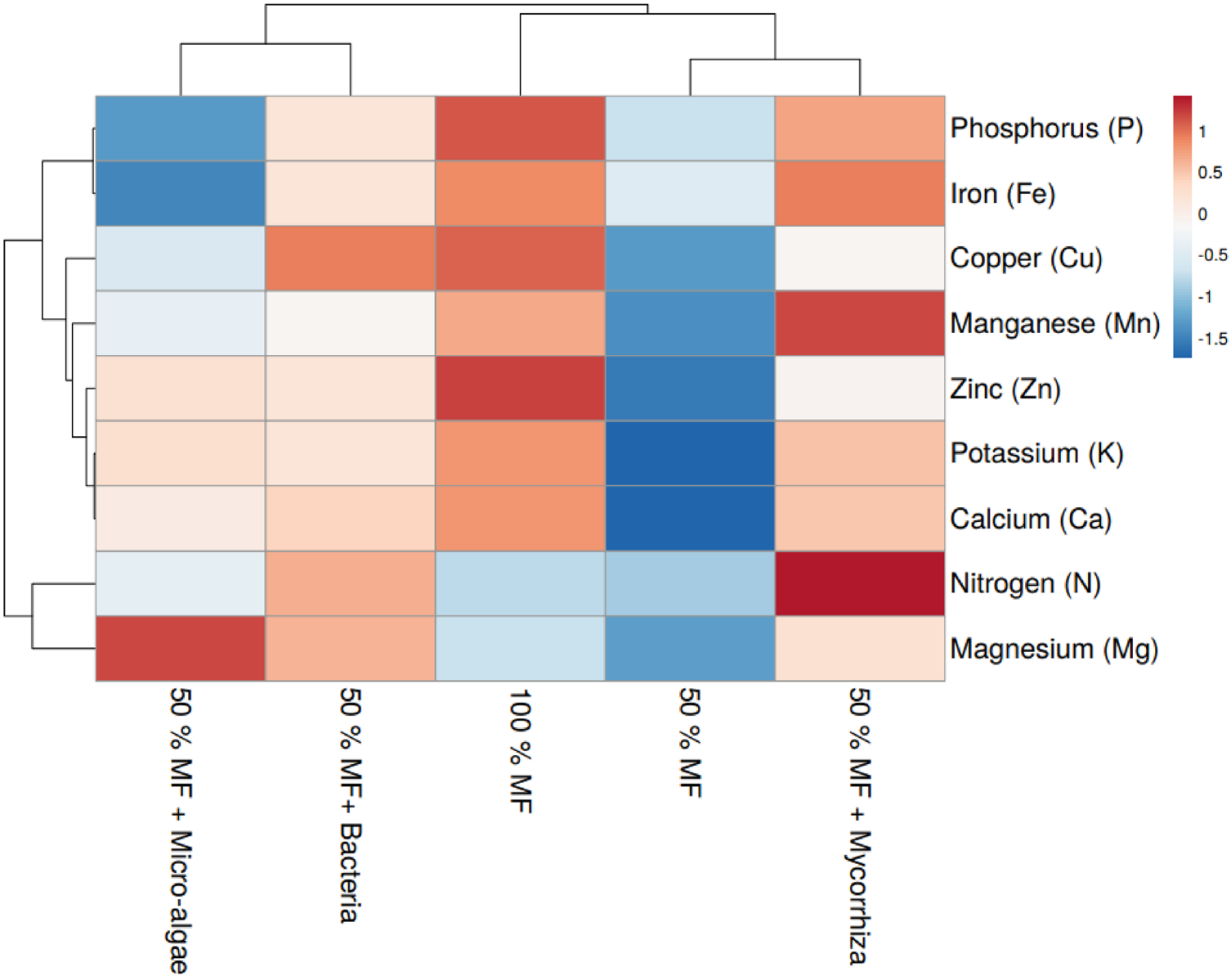
Effect of bio-fertilizers on the concentrations of macro and micro elements in the basil leaf. *MF* Mineral fertilizers.

Biofertilizers can affect the mineral composition of leafy vegetebles by increasing the content of nutrients in terms of health benefits for human.The bio-fertilizers increased the macronutrient concentrations in comparison to 50% control. Mycorrhiza showed the highest amount of azote, phosphorous, potassium, and calcium among the bio-fertilizers (Fig. 2). These agree with results from Guo et al.^40^ with *Glomus* mycorrhiza species that ameliorates phosphorus, nitrogen, and potassium intake. Besides, the colonization of fungi enhances the uptake of P by releasing insoluble phosphorus due to its hyphae^41^. These hyphae can also take up assimilated nitrate, amino acids, and ammonium and transfer the nitrogen to the plant. Mycorrhiza is contributed to N, P, and K uptake by the symbiotic relationship with the host plant^41,42^. Inoculation of mycorrhiza can enhance macro-and micronutrients significantly, leading to increased photosynthate production and biomass accumulation^35^. Begum et al.^35^ indicated that it could lower the use of chemical fertilizers by up to 50% for best agricultural production. However, this estimate depends on the plant species and the prevalent agricultural practices. The highest magnesium was recorded by using microalgae.

### Effect of bio-fertilizers on microelements in basil leaf

The mycorrhiza treatment shows the highest amount of manganese and iron in basil, which are higher than the 100% control (Fig. 2). Several studies confirm that the increase in mineral intake is explained by the formation of an extra radical hyphae chain that enlarges the absorptive area of plant roots, which strengthens the efficiency of the intake of manganese and iron by mycorrhiza^43–45^. In contrast, the highest amount of Cu was recorded by using bacteria. The production of siderophore from certain bacteria, like *Pseudomonas fluorescens*, promotes the accumulation of Cu and Fe due to their ability to regulate the translocation of metals from roots to shoots^46^. Zinc was the abundant element for micro-algae, even higher than 50% control. According to Zaheer et al.^47^, the absorption of zinc by the plant enhances the photosynthesis process, ameliorating the yield and the quality of basil.

### Effect of bio-fertilizes on vitamin C, total phenols and flavonoids contents in basil leaf

Vitamin C values in basil leaves were determined between 64.14 and 47.39 mg/100 g, the highest in 100% mineral fertilizer and the lowest in the bacterial application (Table 6). The microalgae and the mycorrhiza slightly increased by 2.0% and 4.2%, while the bacteria had a 22.3% reducing effect on vitamin C.

**Table 6:** Effects of bio-fertilizers on the vitamin C, total phenols and flavonoids contents in basil leaves grown in floating culture.

Treatments	Vitamin C (mg 100 g FW^−1^)	Total phenols (mg GA/100 g FW)	Total flavonoids (mg RU/100 g FW)
100% mineral fertilizers	64.04^a^	37.04^ab^	41.12^b^
50% mineral fertilizers	61.27^ab^	33.26^c^	42.58^b^
50% mineral fertilizers + micro-algae	62.50^a^	31.92^c^	40.74^b^
50% mineral fertilizers + bacteria	47.39^b^	39.16^a^	57.06^a^
50% mineral fertilizers + mycorrhiza	63.83^a^	33.56^bc^	44.62^b^
LSD_0.05_	2.436	4.89	9.81
*p*-value	0.1226	0.0019	< 0.0001

There is no significant difference between means with the same letter in the same column, *LSD* the least significant difference. *FW* Fresh weight, *GA* Gallic acid, *RU* Rutin.

The highest amount of phenol was found to be 39.16 mg GA/100 g from the bacteria application. The lowest phenol, 31.12 mg GA/100 g, was found in the mico-algae. The mycorrhiza showed a total phenol content of 33.56 mg GA/100 g between the bacteria and the micro-algae applications (Table 6).

Regarding total flavonoids, as in phenol, the bacterial fertilizer gave the highest content with 57.06 mgRU/100 g, while the micro-algae showed the lowest flavonoid content with 40.74 mgRU/100 g. On the other hand, the mycorrhiza was in the middle between the above two applications with 44.62 mgRU/100 g flavonoid content (Table 6). The bacteria were the most effective bio-fertilizer in increasing the high-antioxidant flavonoid in basil leaves. The bacteria had a pronounced enhancing effect on the increase of phenol and flavonoids in the leaves of basil plants. Accordingly, 17.73% phenol and 35.27% flavonoid contents were found to be higher in comparison to 50% mineral nutrients. Babu et al.^52^. indicated that *Bacillus subtilis* and *Bacillu*s *cereus* increased the accumulation of peroxidase and polyphenol oxidase enzymes, which are included in the metabolism of phenols and flavonoids. Phenolics and flavonoids in basil are favourable compounds due to their high antioxidative activities and interest in preserving agents to protect the human body against degenerative diseases caused by oxidative damage^48^. Jiménez-Gómez et al.^49^, reported that the inoculation of plants with different bacteria trains improves flavonoid content compared to the control treatment. The study reported that hydroponic cultivation improved antioxidant activity and increased the contents of vitamin C, total phenols and rosmarinic acid in basil compared to soil-grown ones^50^. The inoculation of basil with a mixture of *Pseudomonas putida* 41, *Azotobacter chroococcum* 5, *Azospirillum lipoferum* strains presented the highest level of antioxidant activity as compared to the control treatment^51^.

## Conclusion

The increase in food production using minimal resources while protecting the environment and human health poses a severe challenge for humanity. Using bio-fertilizers represents a promising and environmentally friendly approach to increasing crop yields and ameliorating quality and antioxidant compounds with fewer resources. This fits very well with the UN Sustainability Goal 12: “Ensure sustainable consumption and production patterns”. An application of bio-fertilizers in hydroponic cultivation of basil cv. ‘Dino’ reduced the need for mineral fertilizers. At the same time, bio-fertilizers affected an increased plant yield and improved product quality. Mycorrhiza was more effective in winter, late autumn, and the beginning of spring. Bacterial bio-fertilizers can be used successfully in warm periods, such as late spring and summer. In *Chlorella vulgaris* case, the mineral fertilisers could be reduced by more than 50% due to possible competition for mineral fertilizers from microalgae.

## Data Availability

All the relevant data have been provided in the manuscript. The corresponding author may provide additional details upon reasonable request.
